# Pharmacist attitudes and provision of harm reduction services in North Carolina: an exploratory study

**DOI:** 10.1186/s12954-021-00517-0

**Published:** 2021-07-08

**Authors:** Rachel A. Parry, William A. Zule, Christopher B. Hurt, Donna M. Evon, Sarah K. Rhea, Delesha M. Carpenter

**Affiliations:** 1 UNC Eshelman School of Pharmacy, 201 Pharmacy Lane, CB 7355, Chapel Hill, NC 27599-7355 USA; 2 RTI International, 3040 East Cornwallis Rd., PO Box 12194, Research Triangle Park, NC 27709-2194 USA; 3 Institute for Global Health and Infectious Diseases, University of North Carolina at Chapel Hill, 130 Mason Farm Rd, CB#7030, Chapel Hill, NC 27599-7030 USA; 4 Division of Gastroenterology and Hepatology, Department of Medicine, University of North Carolina at Chapel Hill, Chapel Hill, NC USA

**Keywords:** Harm reduction, Substance abuse, Intravenous, Community pharmacy services, Syringes, Naloxone, HIV, Hepatitis C

## Abstract

**Background:**

Pharmacists are among the most accessible healthcare providers in the United States and uniquely positioned to provide harm reduction services. The availability of pharmacy-based harm reduction services and pharmacist attitudes toward delivering these services have been understudied to date. We examine North Carolina (NC) pharmacists’ experiences with and attitudes about harm reduction services and explore differences between rural and urban pharmacists.

**Methods:**

A convenience sample of NC pharmacists participated in an anonymous, online survey regarding harm reduction services: non-prescription syringe sales; naloxone dispensing; and human immunodeficiency virus (HIV) and hepatitis C virus (HCV) screening. Urban–rural differences were analyzed using Pearson’s chi-square or Fisher’s exact tests. Open-ended responses were analyzed thematically.

**Results:**

Three hundred pharmacists responded to the survey; 68 (23%) practiced in rural counties. Dispensing non-prescription syringes and naloxone at least occasionally was reported by 77% (*n* = 231) and 88% (*n* = 263) pharmacists, respectively. Pharmacy-delivered HIV or HCV screening was rare. Urban pharmacists dispensed naloxone more frequently than rural pharmacies (*p* = 0.04). Only 52% of pharmacists agreed that persons who inject drugs should always be allowed to buy non-prescription syringes. Rural pharmacists’ attitudes toward harm reduction services for persons who inject drugs were statistically, though marginally, less supportive when compared to urban pharmacists’ attitudes. The most common barrier to non-prescription syringe access was requiring patients to provide proof of prescription injection medication use, which 21% of pharmacists reported was required by their pharmacy’s policy on non-prescription syringe sales.

**Conclusions:**

Although most pharmacies distributed naloxone and sold non-prescription syringes, pharmacy store policies and personal beliefs inhibited naloxone and non-prescription syringe dispensing. NC community pharmacies infrequently offer HIV and HCV screening. Paired with disseminating the evidence of the positive impact of harm reduction on individual and public health outcomes to NC pharmacists, institutional and systems changes to practice and policy may be important to promote harm reduction service availability, particularly for rural NC residents.

Trial registration: N/A.

**Supplementary Information:**

The online version contains supplementary material available at 10.1186/s12954-021-00517-0.

## Background

Over 90% of Americans live within five miles of a community pharmacy (i.e., a pharmacy that dispenses medications to patients for use at their home), making pharmacists among the most accessible healthcare providers in the United States (US) [1]. Pharmacists are particularly important in rural areas where access to other healthcare professionals is limited [2]. Given this accessibility, pharmacists are uniquely positioned to improve public health through offering harm reduction services. Harm reduction (HR) refers to a package of evidence-based interventions that help mitigate individual and community health risks associated with drug use. Such interventions include syringe exchanges, selling syringes without a prescription, dispensing naloxone (an opioid overdose reversal agent), and screening for human immunodeficiency virus (HIV) and hepatitis C virus (HCV). HR services have been found to be a cost-effective intervention to avert HIV and/or HCV infections [3].

The availability of non-prescription syringes (NPS) in the US has a rich history. In response to rising opiate addiction in the early 1900s, state laws began restricting access to hypodermic needles and syringes to curb illicit drug use [4]. In response to the HIV epidemic, beginning in the 1980s, some states relaxed those restrictions in an effort to increase availability of sterile syringes and reduce HIV transmission [4]. North Carolina (NC) law currently allows pharmacists to sell syringes without a prescription, but NPS sales occur at pharmacist discretion [5, 6]. Therefore, pharmacist attitudes and pharmacy policies have a large influence on NPS availability. Over 50% of pharmacists surveyed in Indiana in 2016 reported feeling uncomfortable dispensing syringes to anyone without a prescription [7]. Negative experiences related to NPS sales, such as discovering used syringes on the store floor or persons who inject drugs (PWID) refusing to wait their turn in line during busy times, were reasons Connecticut pharmacists refused to sell NPS [8]. Notably (and paradoxically, given ample evidence on the benefits of NPS), pharmacies located in areas with high rates of opioid overdose mortality are often less likely to sell syringes without a prescription [7]. PWID report that pharmacies are where they most frequently purchase sterile syringes, and those residing in urban settings may have easier access to sterile syringes than those in rural areas [9].

In 2015, the rate of drug overdoses was higher in non-metropolitan (17.0 per 100,000 population) than in metropolitan areas (16.2 per 100,000) – a reversal of prior trends [10]. Naloxone, an opioid antagonist, can reverse the fatal effects of opioid overdose [11]. In an effort to decrease opioid-associated mortality, all 50 states and the District of Columbia have passed laws to increase communities’ access to naloxone through pharmacy dispensing [12]. Increasing training and access to naloxone use and supplies to individuals at risk of opioid overdose has been associated with decreased opioid-related deaths, such as a Scottish study that demonstrated a 36% reduction in opioid-related deaths following implementation of a naloxone program [13].

In NC, pharmacists are allowed to dispense naloxone to patients at risk of opioid overdose or anyone in a position to help overdose victims under a statewide order issued by the State Health Director in 2016 [14]. Due to the relatively recent passage of naloxone dispensing laws, fewer pharmacies participate in distribution of naloxone than in NPS sales [15]. In 2018, naloxone dispensing rates were lower in rural counties (147.4 naloxone prescriptions per 100,000 population) versus either micropolitan (206.3 naloxone prescriptions per 100,000 population) or metropolitan (169.1 naloxone prescriptions per 100,000 population) counties [16]. Rural counties also had the lowest rates of naloxone prescriptions per number of high-dose opioids dispensed, with rural counties dispensing 0.43 naloxone prescriptions per 100 high-dose opioids, micropolitan counties dispensing 0.53 naloxone prescriptions per 100 high-dose opioids, and metropolitan counties dispensing 0.57 naloxone prescriptions per 100 high-dose opioids [16]. Lower naloxone dispensing is not accounted for by a difference in need alone, and the Centers for Disease Control and Prevention (CDC) notes that pharmacies’ dispensing of naloxone can benefit rural communities where HR services are often more scarce [16]. Remaining barriers to provision of naloxone include pharmacist time constraints, lack of pharmacist reimbursement for service provision, ethical concerns about drug abuse, and concerns about clientele who would come to the pharmacy [17, 18].

HIV and HCV screening through point-of-care tests can be offered and billed by pharmacists in community pharmacy settings, provided staff have been trained to perform the test and secured permission from the federal government to administer tests outside of a formal clinical laboratory setting [19]. As of January 24, 2020, 430 pharmacies in North Carolina had been granted this permission [20]. The feasibility, successful follow-up, and positive perception among pharmacy staff and patients of pharmacist-provided HIV and HCV screening and further referral (as medically appropriate) has been demonstrated in prior work [21–24]. Barriers to initiating these screening interventions include pharmacist time constraints and connecting with at-risk patients who are not already linked to care in the pharmacy [24]. A Virginia study found that retail pharmacies may be useful for increasing community access to HIV testing, especially when the pharmacies are located in areas with few other testing resources [25].

Rural areas in the US have fewer syringe service programs (programs which offer sterile syringes and often other services such as free HIV testing) than urban areas; therefore, PWID living in rural areas have less access to HR services than their non-rural counterparts [26]. As one of the most accessible healthcare professionals, pharmacists could alleviate this disparity by consistently offering HR services for their communities [1]. However, little is known about the uptake and attitudes toward HR strategies in rural community pharmacies. One study in Kentucky found no significant difference in pharmacists’ willingness to provide sterile syringes and needles between rural and urban pharmacies but did not analyze attitudes toward this service [27]. Furthermore, the impact of attitudes among pharmacists working in rural-based practices may be magnified, as access to HR services is more scarce in rural areas, in general. This study seeks to understand urban–rural differences in the provision of and pharmacist attitudes toward four HR services: selling syringes without a prescription; distributing naloxone; HIV screening; and HCV screening.

## Methods

### Procedure

An email was sent in May 2019 with a link to an anonymous, online survey to all pharmacists with an active license registered with the North Carolina State Board of Pharmacy (NCBOP) using the NCBOP listserv. A two-week reminder email was sent, and the survey was open for one month.

Pharmacists answered one eligibility screening question before beginning the survey to confirm that they currently practiced in a community pharmacy. Eligible pharmacists advanced to the survey, which took approximately 5–10 min to complete. No incentive was offered to participate. Data were collected using Qualtrics software (Qualtrics, Provo, UT). This study received an exemption from the University of North Carolina Institutional Review Board.

### Measures

#### Demographics

Demographics and experiential history of the pharmacist were collected, including the following variables: age group; gender; racial/ethnic background; highest level of pharmacy education; type of community pharmacy; position in pharmacy; time practicing as a pharmacist; and time practicing in current pharmacy.

#### Rurality

Rurality was assessed using the Rural–Urban Continuum Codes (RUCC) developed by the United States Department of Agriculture (USDA) [28]. There are 9 RUCC categories, ranging from 1 (counties in metropolitan areas with populations of at least 1 million) to 9 (counties not adjacent to a metropolitan area that are completely rural or have an urban population of less than 2500). Using a map of North Carolina, each county was color-coded by RUCC level, and participants selected the color of the county in which their pharmacy was located. Based on prior literature and the USDA Economic Research Service recommendation for urban/rural dichotomization for studies with a purpose such as ours, the nine rural–urban continuum codes were dichotomized into two categories: metropolitan/urban (RUCC 1-3) and non-metropolitan/rural (RUCC 4-9) [29–33].

#### Non-prescription Syringe Sales

Participants were asked if their pharmacy has a policy about the sale of NPS to customers, and if so, to describe it. They also were asked how often they sell NPS (never; occasionally, but less frequently than once per month; on average, once per month; 2–3 times per month; weekly; daily; or, my pharmacy does not sell NPS). Separately, participants were asked if they had ever refused to sell NPS at their pharmacy, and if those that had refused were asked via an open-ended question to specify their reason for refusing. Participants also reported their willingness to sell NPS on a 5-point Likert scale (1 = “very unwilling” to 5 = “very willing” with “undecided” at the midpoint) to the following types of customers: (1) patients with diabetes; (2) persons suspected of injecting drugs; and (3) PWID who have a referral to purchase syringes, for example, a formal referral from a harm reduction organization [34]. For analyses, responses were dichotomized into “willing” (“somewhat willing” or “very willing”) versus “not willing” (“very unwilling,” “somewhat unwilling,” or “undecided”).

#### Naloxone

Participants were asked if they have ever dispensed naloxone. If yes, they reported how often and to whom. Participants also reported their willingness to dispense naloxone on a 5-point Likert scale (1 = “very unwilling” to 5 = “very willing” with “undecided” at the midpoint) to the following customers: (1) patient with naloxone co-prescription from a physician (i.e. where a prescriber writes two prescriptions during one patient encounter: one prescription for an opioid and the other for naloxone); (2) persons suspected of injecting drugs; (3) third parties (like parents or friends) of individuals who may be at increased risk of opioid overdose; and (4) patient with an opioid prescription (without a naloxone co-prescription). For analyses, responses were dichotomized into “willing” (“somewhat willing” or “very willing”) versus “not willing” (“very unwilling,” “somewhat unwilling,” or “undecided”). We also asked if participants were interested in learning more about naloxone, and if so, to indicate which naloxone topics they were interested in learning about.

#### HIV and HCV Screening

Participants were asked if they offer HIV or HCV screenings at their pharmacy and what HIV and HCV educational topics they would be interested in learning more about.

#### Pharmacist attitudes toward persons who inject drugs

We asked participants to rate their level of agreement with 14 statements about HR service provision to PWID. Response options were on a 5-point Likert scale from 1 = “strongly disagree” to 5 = “strongly agree” with a midpoint of “no opinion.” These attitude statements came from a previously published survey about pharmacist attitudes and beliefs regarding sales of NPS and included the following sections: whether PWID should be allowed to buy NPS; negative influences of PWID on other customers and pharmacy safety; and pharmacist role in providing PWID healthcare resources, including resources for HIV prevention [22]. The scale demonstrated good internal consistency (Cronbach’s *α* = 0.86), supporting reliability of the measure.

### Data Analysis

Quantitative data were analyzed using SAS 9.4® (SAS Institute, Cary, NC). Summary tables were created to examine descriptive statistics, and Pearson’s chi-square and Fisher’s exact tests were used to evaluate differences by rurality at *α* = 0.05. Responses from the two open-ended questions (describing the pharmacy’s policy regarding NPS sales and the reason they refused to sell NPS) were imported into Microsoft Excel and first analyzed using inductive coding by two of the authors [35]. The coders then met to discuss their respective codes and refine them to create a final coding scheme. Themes were organized into a structured codebook, which included example quotations and definitions. Each coder independently reread all open-ended responses and identified the themes present in each response, after which the coders met and resolved discrepancies through consensus. The number of pharmacists who mentioned each theme was totaled.

## Results

### Demographics

The recruitment email was successfully delivered to 11,609 pharmacists on the NCBOP email listserv. Of those, 458 pharmacists began the survey, but 152 were ineligible because they were not a pharmacist (*n* = 1) or were not practicing in a community setting (*n* = 151). Pharmacists were excluded from analysis if they did not answer the rurality question (*n* = 6). The final sample consisted of 300 pharmacists who practiced at independent (36%) and national chains (45%). The majority held a doctor of pharmacy (PharmD) degree (66%), were female (68%), White (93%), younger than 45 years (56%), and practicing in urban counties (77%; Table 1).

**Table 1:** Pharmacist demographics

Demographic	Urban (*n* = 232)	Rural (*n* = 68)
	% (*n*)	% (*n*)
Community pharmacy type		
Independent	31.9 (74)	48.5 (33)
National chain	47.4 (110)	36.8 (25)
Regional or local chain or grocery store pharmacy	9.9 (23)	8.8 (6)
Health-system affiliated outpatient, including VA	7.3 (17)	1.5 (1)
Community health center or charity	1.3 (3)	4.4 (3)
State Facility	1.7 (4)	0 (0)
Specialty Pharmacy	0.4 (1)	0 (0)
Pharmacist role		
Manager/owner	38.8 (90)	50.0 (34)
Staff, resident, or fellow	59.9 (139)	42.7 (29)
Relief, float, or part-time	1.3 (3)	7.4 (5)
Years practicing as a pharmacist		
< 11	40.1 (93)	41.2 (28)
11–30	40.1 (93)	35.3 (24)
> 30	19.4 (45)	23.5 (16)
No response	0.4 (1)	0 (0)
Years at this pharmacy		
< 1	13.8 (32)	16.2 (11)
1–5	49.1 (114)	38.2 (26)
6–10	12.1 (28)	14.7 (10)
11–20	16.0 (37)	20.6 (14)
> 20	6.0 (14)	6.6 (6)
No response	3.0 (7)	1.5 (1)
Highest level of pharmacy education		
BSPharm	31.9 (74)	35.3 (24)
MSPharm	0.4 (1)	0 (0)
PharmD	67.2 (156)	64.7 (44)
Other	0.4 (1)	0 (0)
Age		
25–34	31.5 (73)	35.3 (24)
35–44	25.4 (59)	19.1 (13)
45–54	17.24 (40)	16.2 (11)
55–64	14.7 (34)	17.7 (12)
65–84	4.3 (10)	4.4 (3)
No response or prefer not to answer	6.9 (16)	7.4 (5)
Gender		
Male	28.9 (67)	38.2 (26)
Female	70.3 (163)	61.8 (42)
Other identity	0.9 (2)	0 (0)
Racial/ethnic background (select all that apply)		
White or caucasian	92.7 (215)	92.7 (63)
Black or African American	3.0 (7)	2.9 (2)
American Indian or Alaska Native	0.9 (2)	1.5 (1)
Asian	4.3 (10)	2.9 (2)
Hispanic or latino	1.7 (4)	1.5 (1)
Other	0.4 (1)	0 (0)

### Non-Prescription Syringe (NPS) Sales

Twenty-seven (9%) pharmacists reported that their pharmacies do not sell NPS, and a higher percentage of rural pharmacists (49%) reported past history of refusing to sell NPS compared to urban pharmacists (38%; *χ*^2^ = 2.12, *df* = 1, *p* = 0.15; Table 2). Pharmacists reported selling NPS daily (25%), weekly (17%), 2–3 times per month (14%), once per month (5%), less frequently than once per month (15%), and never (8%). The majority of pharmacists (72%) reported never talking about HR services with customers who bought NPS.

**Table 2:** Pharmacist experience with harm reduction services

Service and experience	Urban (*n* = 232)	Rural (*n* = 68)	*p*-value
	% (*n*)	% (*n*)	
**Non-prescription syringes**			
Does your pharmacy have a policy regarding the sale of non-prescription syringes?			
Yes	43.5 (101)	52.9 (36)	0.08
No	46.1 (107)	38.2 (26)	
Not sure	4.7 (11)	0 (0)	
Missing	5.6 (13)	8.8 (6)	
How often do you sell non-prescription syringes at your pharmacy?			
Never	8.6 (20)	4.4 (3)	0.56
Occasionally, but less frequently than once per month	13.8 (32)	19.1 (13)	
On average, once per month	4.7 (11)	5.9 (4)	
2–3 times per month	14.7 (34)	13.2 (9)	
Weekly	16.0 (37)	22.1 (15)	
Daily	26.3 (61)	22.1 (15)	
My pharmacy does not sell non-prescription syringes	9.9 (23)	5.9 (4)	
Missing	6.0 (14)	7.4 (5)	
Have you ever refused to sell non-prescription syringes to a customer at your pharmacy?			
Yes	37.9 (88)	48.5 (33)	0.15
No	46.1 (107)	38.2 (26)	
Missing	16.0 (37)	13.2 (9)	
How often do you talk about harm reduction with customers to whom you sell non-prescription syringes?			
Never	59.9 (139)	60.3 (41)	0.87
Sometimes	19.4 (45)	19.1 (13)	
Often	2.6 (6)	4.4 (3)	
Always	1.7 (4)	1.5 (1)	
Missing	16.4 (38)	14.7 (10)	
**Naloxone**			
How often do you dispense naloxone at your pharmacy?			
Occasionally, but less frequently than once per month	41.0 (95)	54.4 (37)	0.04
On average, once per month	21.6 (50)	7.4 (5)	
2–3 times per month	18.1 (42)	17.7 (12)	
Daily or weekly	7.8 (18)	5.9 (4)	
Missing	11.6 (27)	14.7 (10)	
Who have you dispensed naloxone to?*			
Patients	85.8 (199)	82.4 (56)	0.49
Caregivers	42.7 (99)	33.8 (23)	0.19
First responders	10.3 (24)	7.4 (5)	0.46
Institutions	0.4 (1)	1.5 (1)	0.40
**HIV and HCV**			
Is HIV testing offered at your pharmacy?			
Yes	2.2 (5)	2.9 (2)	0.66
No or unknown	93.5 (217)	91.2 (62)	
Missing	4.3 (10)	5.9 (4)	
Is hepatitis C screening offered at your pharmacy?			
Yes	1.7 (4)	2.9 (2)	0.62
No or unknown	93.1 (216)	91.2 (62)	
Missing	5.2 (12)	5.9 (4)	

*Select all that apply

*p*-values were calculated using Pearson’s chi square and Fisher’s exact tests

Almost half (46%) of pharmacists reported that their pharmacy has a policy regarding NPS sales (described in Additional file 1: Table S1); the most frequently reported policy was a requirement for proof of medical necessity to sell NPS (i.e., prescription injection medication use, 21%). For example, one pharmacist wrote, “no syringes without insulin script,” while another said that the customer “must show proof of medical use (medication vial, etc.)” in order to receive NPS. One pharmacist explained that the store policy required proof of medical necessity and added, “Not my belief but policy has been in place since I moved to this store.” Twenty-two pharmacists (7%) indicated that their pharmacy had no obvious restrictions for NPS sales.

More pharmacists were willing to sell NPS to patients with diabetes (82%), compared to PWID with a referral to purchase syringes (59%), or persons suspected of injecting drugs (49%; Table 3). Willingness to sell NPS differed significantly by rurality for only one specified population: significantly more urban pharmacists (62%) than rural pharmacists (51%) were willing to sell to PWID who have a referral to purchase syringes (*p* < 0.05).

**Table 3:** Pharmacist willingness to engage in harm reduction services for specified populations

	Urban (*n* = 232)	Rural (*n* = 68)	Urban/rural *p*-value
	% (*n*)	% (*n*)	
Willingness to sell non-prescription syringes to:^a^			
Patients with diabetes			
Willing	81.5 (189)	82.4 (56)	0.66
Not willing	2.2 (5)	2.9 (2)	
Suspected persons who inject drugs			
Willing	51.7 (120)	41.2 (28)	0.07
Not willing	31.9 (74)	44.1 (30)	
Persons who inject drugs who have a referral to purchase syringes			
Willing	61.6 (143)	51.2 (35)	0.049
Not willing	22.0 (51)	33.8 (23)	
Willingness to dispense naloxone to:^b^			
Patient with naloxone co-prescription from physician			
Willing	99.6 (224)	94.1 (64)	1.00
Not willing	1.3 (3)	1.5 (1)	
Suspected persons who inject drugs			
Willing	84.1 (195)	79.4 (54)	0.57
Not willing	13.8 (32)	16.2 (11)	
Third parties of individuals who may be at increased risk of opioid overdose			
Willing	90.1 (209)	85.3 (58)	0.47
Not willing	7.8 (18)	10.3 (7)	
Patient with an opioid prescription			
Willing	93.5 (217)	89.7 (61)	0.52
Not willing	4.3 (10)	5.9 (4)	

^a^Willingness to sell non-prescription syringes was missing for 16.4% (*n* = 38) of urban pharmacists and 14.7% (*n* = 10) rural pharmacists

^b^Willingness to dispense naloxone was missing for 2.2% (*n* = 5) urban pharmacists and 4.4% (*n* = 3) rural pharmacists

Many pharmacists declined to sell NPS to a customer at least once (40%). One-hundred and twelve (37%) pharmacists described at least one reason for why they refused to sell NPS (Additional file 1: Table S2), frequently citing refusal to sell because of unverifiable proof of medical necessity (11%). Examples of these responses include, “Could not prove to be used for legitimate use” and “Customer did not have an injectable medication on profile.” Pharmacists also indicated refusing to sell because of concern about on-site drug use (2%). For example, one pharmacist noted, “We don’t sell them because we have had issues with exposed needles left in the store and in the parking lots and bathrooms. Puts us and others at risk.” Pharmacists also referred to inappropriate behavior (2%); for example, refusing to sell NPS because the “patient was verbally abusive.” Another pharmacist noted that when they enforced a minimum or maximum package size of NPS sale requirement (such as only selling NPS by the box or selling no more than one box of NPS at a time), the “customer then told me that they hope I get *(expletive)* murdered.”

### Naloxone

Thirty-two pharmacists (11%) reported never dispensing naloxone from their pharmacy, which did not differ by rurality (*p* = 0.91). Pharmacists most commonly dispensed naloxone less than once per month (44%) and to patients (85%; Table 2). The distribution of how often pharmacists dispensed naloxone significantly differed by rurality, with urban pharmacists dispensing naloxone more frequently than rural pharmacists (*χ*^2^ = 8.31, *df* = 3, p = 0.04).

Pharmacist willingness to dispense naloxone to various customers did not differ significantly between rural and urban pharmacists (Table 3). Most pharmacists were willing to dispense naloxone to patients with a naloxone co-prescription from a physician (96%). Fewer pharmacists were willing to dispense naloxone to suspected PWID (83%). Pharmacists were most interested in learning more about how to start a conversation with patients and caregivers about naloxone (53%) and providing care in a non-stigmatizing way (43%; Additional file 1: Table S3).

### HIV and HCV Screening

In-pharmacy HIV and HCV tests were each offered by 2% of pharmacists. Approximately 8% of pharmacists whose pharmacies were not currently offering these services were interested in offering one or both. There were no significant differences between rural and urban pharmacist provision of HIV (*p* = 0.65) or HCV screening (*p* = 0.62; Table 2). Pharmacists were most interested in learning about treatment for HIV (49%), options for offering HIV testing at their pharmacy (44%) and reimbursement options for HIV testing at their pharmacy (44%; Additional file 1: Table S3).

### Pharmacist attitudes toward persons who inject drugs (PWID)

A larger percentage of pharmacists agreed that providing NPS is a safe method of preventing blood-borne infections among PWIDs (76%), but fewer agreed that PWID should always be allowed to buy NPS (52%).

Attitudes toward PWID significantly differed statistically for four of the 14 statements (Table 4). First, urban pharmacists (mean 3.5, SD 1.41) more strongly agreed than rural pharmacists (mean 3.09, SD 1.49) that, “PWIDs should always be allowed to buy NPS,” (*pooled t-*test =  − 2.02, *p* = 0.04). Urban pharmacists also more strongly agreed (mean 4.2, SD 1.06) than rural pharmacists (mean 3.72, SD 1.3) that, “Providing NPS is a safe method of preventing blood-borne infections among PWIDs,” (*Satterthwaite t-*test = 2.64, *p* < 0.01). Rural pharmacists (mean 2.58, SD 1.41) more strongly agreed (as indicated by being closer to the scale’s midpoint of “No opinion”) than urban pharmacists (mean 2.09, SD 1.07) that, “If I could, I would refuse to treat PWID customers”, (*Satterthwaite t-*test = 2.53, *p* = 0.01). Urban pharmacists (mean 2.63, SD 1.03) more strongly disagreed than rural pharmacists (mean 2.93, SD 1.13) that, “It is not the role of the pharmacists/pharmacy staff to provide PWID customers with HIV prevention services,” (*pooled t-test* = 1.98, *p* < 0.05).

**Table 4:** Pharmacist Attitudes/agreement with the following statements

Statement	Mean ± SD		*p*-value (*t*-test)
	Urban	Rural	
Persons who inject drugs (PWIDs) should always be allowed to buy non-prescription syringes^a,e^	3.50 ± 1.41	3.08 ± 1.49	0.04*
Providing non-prescription syringes is a safe method of preventing blood-borne infections among PWIDs^a,e^	4.20 ± 1.06	3.72 ± 1.30	0.01*
PWID customers are a disruption to my pharmacy^a,e^	2.98 ± 1.24	3.30 ± 1.31	0.08
If I could, I would refuse to treat PWID customers^b,e^	2.09 ± 1.07	2.58 ± 1.41	0.01*
PWID customers make other customers feel uncomfortable^b,e^	3.38 ± 1.15	3.68 ± 1.05	0.06
PWID customers make my pharmacy less safe^b,e^	3.14 ± 1.22	3.47 ± 1.20	0.06
Pharmacists/pharmacies are an important resource for PWIDs who may not be able to access health care in the community^b,e^	3.95 ± 1.04	3.67 ± 1.17	0.07
Pharmacists should provide HIV prevention information/resources to PWIDs who purchase non-prescription syringes^b,e^	3.57 ± 1.01	3.43 ± 1.28	0.45
Pharmacists should provide HIV prevention information/resources to anyone who purchases non-prescription syringes^c,e^	3.17 ± 1.17	2.97 ± 1.09	0.23
It is not the role of the pharmacists/pharmacy staff to provide PWID customers with HIV prevention services^c,e^	2.63 ± 1.03	2.93 ± 1.13	0.049*
I am willing to provide information/resources to PWID customers who purchase non-prescription syringes^d,e^	3.85 ± 0.93	3.65 ± 1.04	0.15
I have time to provide information/resources to PWID customers who purchase non-prescription syringes^d,e^	2.58 ± 1.23	2.33 ± 1.27	0.18
I am concerned about mistaking people purchasing non-prescriptions syringes as drug users^d,e^	3.32 ± 1.19	3.18 ± 1.37	0.49
I would support a syringe disposal receptacle on the premises of my pharmacy^d,e^	3.09 ± 1.32	3.02 ± 1.48	0.69

Mean and standard deviation calculated on likert scale where 1 = strongly disagree, 5 = strongly agree, with midpoint 3 = no opinion

^*^Significant at *α* = 0.05

^a^Level of agreement with this statement was missing from 7.8% (*n* = 18) of urban pharmacists

^b^Level of agreement with this statement was missing from 8.2%% (*n* = 19) of urban pharmacists

^c^Level of agreement with this statement was missing from 8.6% (*n* = 20) of urban pharmacists

^d^Level of agreement with this statement was missing from 9.1% (*n* = 21) of urban pharmacists

^e^Level of agreement with this statement was missing from 11.8% (*n* = 8) of rural pharmacists

## Discussion

We found several urban–rural differences in pharmacists’ HR experience and attitudes, though the clinical significance of such differences should be considered. Rural pharmacists reported dispensing naloxone less frequently than urban pharmacists, and fewer rural pharmacists were willing to sell NPS with a physician’s referral than urban pharmacists. There were no differences between urban and rural pharmacists in how often they offered HIV testing and HCV screening, which was low regardless of rurality. Rural pharmacists had marginally more negative attitudes toward HR than urban pharmacists.

Over a third of urban pharmacists and almost half of rural pharmacists reported that they had refused to sell NPS to customers. Pharmacists reported refusing to sell NPS to suspected PWID or clients who could not prove that they had a medical indication for a syringe, which aligns with previous work that found pharmacists often sell syringes only to specific patients, such as those who were diabetic [36]. This finding is alarming; pharmacist reluctance to sell to PWID can decrease access to NPS for PWID, for whom NPS programs were designed to reduce transmission of blood-borne pathogens, such as HIV [4]. Expriences with refusal to sell NPS to suspected PWID are not limited to pharmacists in NC or the US. In a series of feasibility studies examining expanded pharmacy services for PWID, many pharmacists in Mexico and Russia also refuse to sell NPS to mystery shoppers who posed as suspected PWID [37]. The experience of being denied NPS from pharmacists can prompt feelings of shame and stigmatization in PWID, discouraging them from seeking sterile NPS from pharmacies in the future [38].

The responsibility to increase access to NPS should not be placed on individual pharmacists alone, as the institutions and systems they work within may discourage NPS sales to PWID. Many pharmacists reported store policies that required proof of medical necessity (i.e. proof of prescription injection medication) in order to purchase NPS. Laws and policies supporting PWID access to NPS in pharmacies are strong motivators for pharmacists to sell NPS, but the presence of structural barriers in NC make it impossible to determine whether pharmacists primarily refused to sell NPS due to personal beliefs or store policy [39]. Asking pharmacists to sell NPS to anyone, regardless of medical necessity, without addressing institutional barriers is unlikely to have the desired results. A multi-faceted approach will be needed in order to increase PWID access to NPS at pharmacies.

Pharmacists’ willingness to dispense naloxone to patients with a valid prescription aligns with prior literature [40]. More pharmacists were more willing to dispense naloxone than they were willing to sell NPS to patients with diabetes, suspected PWID, or customers with a referral for NPS. As pharmacist willingness to dispense naloxone was generally high, interventions should target known barriers to naloxone access, such as the out-of-pocket cost for patients, to reduce opioid-related deaths [17]. As barriers are addressed, it will be crucial to create continuing education for NC pharmacists that reflects the public health benefits of pharmacist-provided HR services, and particularly that targets misconceptions of PWID among pharmacists who resist evidence-based HR services [39]. As structural restrictions are reduced, education on how to integrate HIV and HCV screening, which is not currently well established, into pharmacy workflow may increase pharmacy-offered HR services, and increase the access to these services for at-risk populations.

Rural pharmacists had marginally more negative attitudes toward PWID. The clinical significance of these differences in attitudes are unclear. One study in North Carolina found more barriers to HIV care in rural areas compared to urban areas, including lack of health care professionals who are adequately trained and competent in HIV care [41]. Therefore, while rural pharmacists’ attitudes were only slightly more negative for a handful of statements, the impact of more negative attitudes among pharmacists may be magnified in rural areas, where access to HR services is more scarce.

Continuing education and interventions to increase pharmacy-offered HR services should focus on rural pharmacists in particular. The majority of pharmacists reported “never” talking about HR services with patients to whom they sell NPS. Rural pharmacists would benefit particularly from education about the positive impact they can have on their community through HR service. Additionally, equipping rural pharmacists with the skills and information to talk about local HR resources for PWID may help them feel more empowered to discuss HR with PWID.

### Limitations

This study has multiple limitations. The generalizability of our findings is limited because we used a convenience sample of NC pharmacists. The results may not be generalizable to all NC pharmacists and especially to other states, where experiences and attitudes may vary. Despite this, the results are valuable and provide a foundation for future studies in other states and regions. We were unable to determine an accurate response rate because the exact denominator is unknown. Pharmacists who read the recruitment email may have seen that they were ineligible because they did not work in a community pharmacy setting and therefore have chosen not to respond. Furthermore, we did not collect information about NC pharmacists registered as working in a community pharmacy setting with the NCBOP at the time, and therefore we do not know what percentage of community pharmacists are captured by this response. Even though the number of respondents for this survey was similar to other studies using this recruitment method, the number of respondents was nevertheless low [42–44]. Our results may only reflect the experiences and attitudes of pharmacists who are most interested in HR services, which could lead to an overestimation of the prevalence of HR services experiences and positive attitudes toward them. Even though the number of respondents was low, we were still able to detect significant differences between urban and rural pharmacists. We assessed rurality using RUCC, which fit our intention to understand the geographic access to HR resources. A notable limitation of RUCC, along with other common measurements of rurality based on geographic boundaries, is that these definitions do not take into account other known factors that contribute to rural health disparities, such as culture, demographics, and economic opportunities [45]. Finally, given the exploratory nature of this study and lack of a priori hypotheses, the statistically significant *p*-value threshold was not adjusted for multiple comparisons. Future research should further explore significant differences by rurality. Although our attitude measure was used previously and demonstrated good internal consistency reliability in the current study, we did not perform a full psychometric evaluation of this measure. Future studies in more representative samples should explore the psychometric properties of the measure.

## Conclusions

Our study suggests multiple opportunities for NC community pharmacists to increase their community’s access to HR services. Interventions to increase HR services in NC community pharmacies will need to target structural barriers, such as store policies, as well as individual pharmacists’ attitudes. Educational trainings for pharmacists that highlight the evidence of HR interventions, opportunities to provide HIV and HCV screening, and the magnified impact of interventions offered by rural pharmacists are needed.

## Supplementary Information

**Additional file 1: Table S1**. Pharmacy’s non-prescription syringe sale policies. **Table S2**. Themes of reasons pharmacists refused to sell non-prescription syringes. **Table S3**. Topics Pharmacists Were Interested in Learning More About.

## Data Availability

The datasets used and/or analyzed during the current study are available from Dr. Delesha Carpenter on reasonable request.

## Abbreviations

PWID: Person/people who inject drugs
NCBOP: North Carolina state board of pharmacy
HIV: Human immunodeficiency virus
HCV: Hepatitis C virus
NPS: Non-prescription syringe(s
