# Supplementary material for: Pharmacist attitudes and provision of harm reduction services in North Carolina: an exploratory study
Source: Harm Reduct J. 2021 Jul 8;18:70. doi: 10.1186/s12954-021-00517-0 (PMC8265050; doi:10.1186/s12954-021-00517-0)
Supplement: Supplementary file 1 — Additional file 1: Table S1. Pharmacy’s non-prescription syringe sale policies. Table S2. Themes of reasons pharmacists refused to sell non-prescription syringes. Table S3. Topics Pharmacists Were Interested in Learning More About. [file 12954_2021_517_MOESM1_ESM.docx]

**Supplemental**

| Table S1: Pharmacy’s non-prescription syringe sale policies | |
| --- | --- |
| Theme | All cohort (n=300)  % (n) |
| Patient must provide proof of medical necessity | 21.3 (64) |
| No obvious restrictions to non-prescription syringe sales | 7.3 (22) |
| Patient must provide identification or proof of their age | 7.3 (22) |
| Pharmacy would sell non-prescription syringes, but only in pre-specified quantities or package sizes | 6.0 (18) |
| Other (e.g., “we are a closed-door pharmacy so we only dispense to patients of our clinic” and “must sign for purchase”) | 3.0 (9) |
| Up to the discretion of individual pharmacist | 2.7 (8) |
| No non-prescription syringe sales | 1.3 (4) |
| Policy differed by whether the customer was known or used their pharmacy | 1.0 (3) |

| Table S2: Themes of reasons pharmacists refused to sell non-prescription syringes | |  |  |
| --- | --- | --- | --- |
| Theme | Urban (n=232)  % (n) | | Rural (n=68)  % (n) |
| Pharmacist could not verify proof of medical necessity | 12.1 (28) | | 8.8 (6) |
| The patient was a suspected PWID or the pharmacist suspected illicit use | 9.1 (21) | | 10.3 (7) |
| Patient/customer could not answer correctly answer questions about medical necessity | 3.5 (8) | | 5.9 (4) |
| Patient must provide identification or to be “old enough” | 3.0 (7) | | 7.4 (5) |
| Store policy does not allow non-prescription syringe sales | 3.9 (9) | | 1.5 (1) |
| The customer/patient was not known by the pharmacist | 3.0 (7) | | 4.4 (3) |
| Concern about on-site injection drug use | 3.0 (7) | | 1.5 (1) |
| Pharmacist would only sell in a particular package size, but patient refused that package size | 2.2 (5) | | 0.0 (0) |
| Customer displayed aggressive or inappropriate behavior | 1.3 (3) | | 2.9 (2) |
| Other (e.g., “All the time” and “Did not yet understand the possible health consequences of refusing to sell syringes” | 0.9 (2) | | 4.4 (3) |

| Table S3: Topics Pharmacists Were Interested in Learning More About | | | |
| --- | --- | --- | --- |
| Topics | Participants (n=300)  % (n) | Urban (n=232)  % (n) | Rural (n=68)  % (n) |
| **Naloxone** |  |  |  |
| How to start a conversation about naloxone with patients and caregivers | 52.7 (158) | 52.6 (122) | 52.9 (36) |
| Providing care in a non-stigmatizing way | 43.3 (130) | 43.1 (100) | 44.1 (30) |
| Legal issues related to dispensing naloxone | 42.3 (127) | 37.9 (88) | 57.4 (39) |
| How to counsel patients and caregivers about naloxone | 41.7 (125) | 41.0 (95) | 44.1 (30) |
| Selecting an appropriate product for a patient based on insurance and patient factors | 38.3 (115) | 36.2 (84) | 45.6 (31) |
| How to administer naloxone | 28.3 (85) | 26.3 (61) | 35.3 (24) |
| How to recognize an overdose | 28.0 (84) | 26.7 (62) | 32.4 (22) |
| Identifying patients who could benefit from naloxone | 19.0 (57) | 17.7 (41) | 23.5 (16) |
| I already know a lot about naloxone and do not need any more training | 12.7 (38) | 13.4 (31) | 10.3 (7) |
| Knowing about naloxone is not very important to my job, so I do not need training | 1.7 (5) | 1.7 (4) | 1.5 (1) |
| Other (e.g., “Support programs for free drugs” and “how to get other pharmacists to understand the importance of Narcan without stigmatization”) | 3.0 (9) | 2.6 (6) | 4.4 (3) |
|  |  |  |  |
| **HIV** |  |  |  |
| Treatment for HIV | 49.0 (148) | 48.3 (112) | 51.5 (35) |
| Options for offering HIV testing at my pharmacy | 44.0 (132) | 47.4 (110) | 32.4 (22) |
| Reimbursement for offering HIV testing at my pharmacy | 44.0 (132) | 46.6 (108) | 35.3 (24) |
| The basics about HIV | 33.0 (99) | 31.5 (73) | 38.2 (26) |
| Prevention of HIV | 26.0 (78) | 23.3 (54) | 35.3 (24) |
| Risk factors for contracting and transmitting HIV | 18.0 (54) | 16.0 (37) | 25.0 (17) |
| AIDS | 16.0 (48) | 13.4 (31) | 25.0 (17) |
| Knowing about HIV is not very important to my job, so I do not need training | 5.0 (15) | 4.7 (11) | 5.9 (4) |
| I already know a lot about HIV and do not need additional training | 4.3 (13) | 3.5 (8) | 7.4 (5) |
| Other (e.g., “Not interested in testing here” and “Refresher would be nice”) | 1.7 (5) | 1.7 (4) | 1.5 (1) |
|  |  |  |  |
| **HCV** |  |  |  |
| Treatment of hepatitis C | 52.0 (156) | 53.0 (123) | 48.5 (33) |
| Drug-drug interactions with hepatitis C medications | 52.0 (156) | 51.3 (119) | 54.4 (37) |
| Differences between screening, confirmatory testing, and hepatitis C antibodies/antigens | 49.3 (148) | 50.4 (117) | 45.6 (31) |
| The basics about hepatitis C | 43.3 (130) | 41.4 (96) | 50.0 (34) |
| Risk factors for contracting and transmitting hepatitis C | 34.7 (104) | 32.8 (76) | 41.2 (28) |
| Prevention of hepatitis C | 34.0 (102) | 31.5 (73) | 42.7 (29) |
| Liver Disease, Cirrhosis, Liver Transplant | 29.3 (88) | 28.5 (66) | 32.4 (22) |
| Knowing about hepatitis C is not very important to my job, so I do not need training | 5.3 (16) | 5.6 (13) | 4.4 (3) |
| I already know a lot about hepatitis C and do not need additional training | 4.0 (12) | 3.9 (9) | 4.4 (3) |
| Other (e.g., “The options available to use for testing” and “screening options and payment options”) | 2.7 (8) | 2.6 (6) | 2.9 (2) |
